# Dual Regulatory Functions and Therapeutic Potential of CD48 in Tumor Immunity

**DOI:** 10.32604/or.2026.082272

**Published:** 2026-07-16

**Authors:** Zhenan Lin, Zhongwu Chen, Zihua Deng, Tingting Bao, Sandi Shen

**Affiliations:** Breast & Thyroid Surgery, the Affiliated Qingyuan Hospital (Qingyuan People’s Hospital), Guangzhou Medical University, Qingyuan, China

**Keywords:** Tumor immunity, CD48, 2B4 (CD244), CD2

## Abstract

Cluster of differentiation 48 (CD48) is a glycosylphosphatidylinositol-anchored member of the signaling lymphocyte activation molecule (SLAM) family that is predominantly expressed on hematopoietic cells and regulates immune-cell communication through 2B4 (CD244) and CD2. This narrative review critically summarizes the context-dependent role of CD48 in tumor immunity, with emphasis on the distinction between activating trans-interactions and potentially inhibitory cis-interactions. Evidence from hematologic malignancies and selected solid tumors indicates that CD48 may support antitumor immunity by facilitating natural killer (NK) cells activation, CD8^+^ T-cell co-stimulation, immune synapse formation, and effector cytokine production. Conversely, loss of CD48 expression, sustained CD48-2B4 engagement, altered ligand density, and suppressive myeloid-rich tumor microenvironment (TME) may contribute to immune escape or NK cells dysfunction. Current therapeutic concepts, including anti-CD48 monoclonal antibodies, antibody-drug conjugates, bispecific antibodies, engineered NK/T cells, and epigenetic restoration of CD48 expression, remain largely preclinical and require cautious interpretation. Major translational barriers include broad CD48 expression on normal hematopoietic populations, soluble CD48 (sCD48) interference, uncertain biomarker standardization, and the risk that forced activation in dense solid tumors may reinforce cis-inhibitory signaling rather than improve cytotoxicity. Future studies should define tumor-type-specific signaling states, quantify sCD48, integrate spatial and single-cell approaches, and evaluate rational combinations with programmed cell death protein 1 (PD-1)/programmed death-ligand 1 (PD-L1) blockade in mechanism-driven models before clinical translation. This review aims to critically evaluate the dual regulatory roles of CD48 in tumor immunity, distinguish between activating trans-interactions and potentially inhibitory cis-interactions across different tumor types, and assess the translational potential and challenges of CD48-targeted immunotherapeutic strategies.

## 1 Introduction

This narrative review (not a systematic review or meta-analysis) selected literature from PubMed and Web of Science on CD48, 2B4 (CD244), CD2, SLAM-family signaling, NK cell biology, tumor immune evasion, immune checkpoints, and CD48-directed therapeutic strategies. Priority was given to mechanistic studies directly involving CD48, tumor-immunity studies, and clinically relevant translational reports. Evidence derived primarily from transcriptomic association analyses or non-CD48 checkpoint literature was interpreted as hypothesis-generating and was not treated as direct mechanistic proof.

### 1.1 Molecular Structure and Biological Functions of CD48

CD48 is a key member of the SLAM family, primarily expressed on the surface of hematopoietic cells, including NK cells, T cells, B cells, and myeloid cells [1,2]. Its function is mainly mediated through binding to the receptor 2B4 (CD244) or CD2, facilitating intercellular signaling and co-stimulatory interactions [3,4]. The engagement between CD48 and 2B4 is critical for NK cell activation: 2B4 recognizes CD48 to induce NK cell cytotoxic responses, thereby promoting immune synapse formation and granule release, which directly contribute to the killing of tumor cells [3,5]. Notably, the activation of this signaling pathway exhibits a density-dependent manner, where the strength of the immune response is co-determined by the expression levels of CD48 and 2B4 on the surfaces of NK cells and target cells [5]. Moreover, CD48 can act synergistically with CD2 to enhance T cell receptor (TCR) signaling, promoting the secretion of interleukin-2 (IL-2) and interferon-gamma (IFN-γ) and driving the differentiation of CD8^+^ T cells into functional effector cells [6]. Recent studies have also revealed that soluble CD48 (sCD48) participates in immune regulation, although its precise mechanisms require further elucidation [2].

Beyond NK- and T-cell-centered signaling, CD48 expression on B cells, monocytes/macrophages, dendritic cells, and hematopoietic progenitor compartments may shape antigen presentation, inflammatory cytokine production, immune-cell clustering, and the availability of 2B4/CD2 ligands within the tumor microenvironment (TME). Therefore, CD48 should not be interpreted as a single-lineage NK-cell activator; rather, its function depends on the cellular source of CD48, the receptor-bearing partner cell, ligand density, and the spatial arrangement of immune and tumor cells. sCD48 is also biologically relevant because it may compete with membrane-bound CD48 for receptor binding or act as a systemic indicator of immune activation, although quantitative cancer-specific thresholds have not yet been established.

### 1.2 Fundamental Framework of Tumor Immune Responses

Tumor immune escape is a core mechanism in cancer progression, involving multiple pathways through which tumor cells suppress immune cell function. These include downregulation of major histocompatibility complex-I (MHC-I) molecules, secretion of immunosuppressive factors such as transforming growth factor-beta (TGF-β), and upregulation of immune checkpoint molecules like programmed death-ligand 1 (PD-L1). Although immune checkpoint inhibitors (ICIs) and chimeric antigen receptor (CAR)-T therapies have achieved breakthroughs in treating certain cancers, their efficacy remains limited by factors such as tumor heterogeneity, the immunosuppressive microenvironment, and treatment resistance [7,8,9]. For example, NK cells infiltrating tumors are susceptible to depletion induced by regulatory cells within the microenvironment (such as tumor-associated macrophages (TAMs)), resulting in functional impairment [8]. Furthermore, conventional immunotherapies often yield suboptimal results in low-immunogenicity tumors (e.g., certain subtypes of non-small cell lung cancer), highlighting the urgent need to identify novel immune regulatory targets [10].

### 1.3 Scientific Significance of CD48 in Tumor Immunity Research

CD48, as a key immunoregulatory molecule in the TME, exhibits a dual regulatory role. On the one hand, high CD48 expression is significantly associated with a favorable prognosis in hematologic malignancies such as acute myeloid leukemia (AML) and multiple myeloma (MM), where it promotes immune surveillance by activating the antitumor activity of NK cells and CD8^+^ T cells [11,12,13,14] Experimental studies have shown that CD48-deficient tumor cells display markedly reduced sensitivity to NK cell-mediated killing, whereas anti-CD48 antibodies can suppress tumor growth by enhancing complement-dependent cytotoxicity (CDC) [12,15]. On the other hand, CD48 may also suppress NK cell activity through cis-interactions, and its expression is dynamically regulated by oncogenes (e.g., promyelocytic leukemia protein/retinoic acid receptor alpha (PML-RARA)) and TGF-β signaling pathways, suggesting its involvement in immune evasion [7,11]. This functional duality positions CD48 as a pivotal node linking immune activation and suppression in tumors. Its expression levels, spatial distribution, and interaction patterns—such as competitive binding with other SLAM family members-collectively influence the direction of the immune response [4,12,16]. In-depth investigation of the CD48 regulatory network will not only facilitate the elucidation of the dynamic balance within the tumor immune microenvironment, but also provides a solid theoretical basis for the development of novel pharmacological strategies such as CD48-targeted antibodies, antibody-drug conjugates, bispecific antibodies, and engineered immune cells [2,7,12,15].

**Figure 1:**
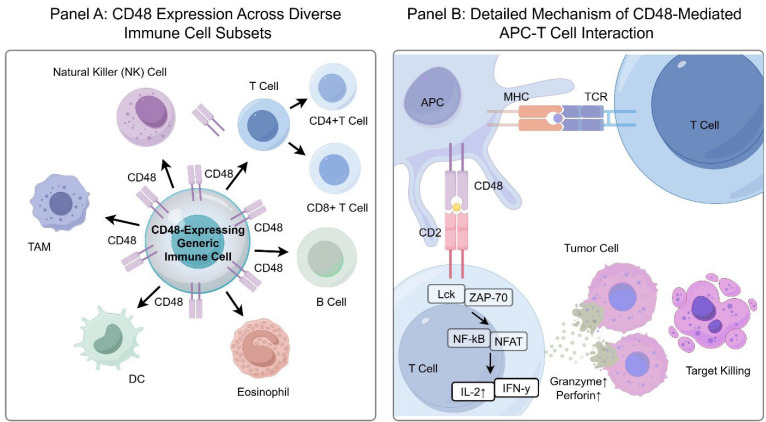
Expression profile of CD48 across immune cell subsets and molecular mechanisms of co-stimulating T cell activation. (**A**) Expression profile of CD48 across diverse immune cell subsets. CD48 is widely expressed on NK cells, T cells (including CD4^+^ and CD8^+^ T cell subpopulations), B cells, eosinophils, DCs, and TAMs. (**B**) CD48-mediated APC-T cell interaction and downstream signaling pathways. During APC-T cell contact, CD48 specifically binds to the CD2 receptor on T cells, serving as a classic co-stimulatory signal that synergistically enhances TCR-MHC complex-mediated immune recognition. This interaction activates downstream Lck and ZAP-70 kinases, thereby driving the nuclear translocation of transcription factors NF-κB and NFAT. This cascade promotes the secretion of IL-2 and IFN-γ, and ultimately upregulates the release of perforin and granzymes to execute targeted tumor cell killing. Abbreviations: APC, Antigen-presenting cell; CD48, Cluster of differentiation 48; DCs, Dendritic cells; IFN-γ, Interferon-gamma; IL-2, Interleukin-2; MHC, Major histocompatibility complex; NFAT, Nuclear factor of activated T cells; NF-κB, Nuclear factor kappa-light-chain-enhancer of activated B cells; NK, Natural killer; TAMs, Tumor-associated macrophages; TCR, T cell receptor. The figure was created using Figdraw (https://www.figdraw.com, accessed on 20 May 2026).

The aim of this review is to systematically evaluate the context-dependent dual regulatory roles of CD48 in tumor immunity, distinguish between activating trans-interactions and potentially inhibitory cis-interactions across different tumor types, and assess the translational potential and major challenges of CD48-targeted immunotherapeutic strategies (Fig. 1).

## 2 Regulatory Role of CD48 in Tumor Development and Progression

### 2.1 CD48-Mediated Mechanisms of Immune Evasion

**Figure 2:**
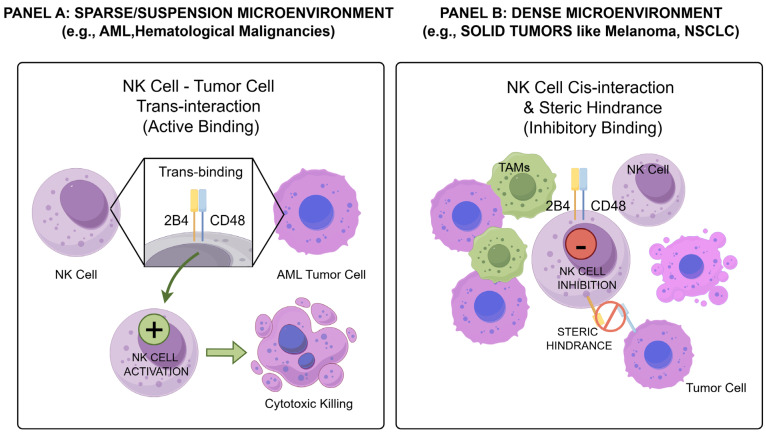
Topological architecture of the TME dictates the Cis/Trans equilibrium and immune outcomes of the CD48-2B4 Axis. (**A**) Trans-activation mode in loose/suspension microenvironments (e.g., hematological malignancies). In hematological malignancies such as AML, tumor cells and effector cells are distributed in a loose, suspension state. This configuration favors the intercellular trans-binding between CD48 on tumor cells and the 2B4 receptor on NK cells, efficiently transducing positive immunoactivating signals and triggering NK cell-mediated cytotoxicity. (**B**) Cis-inhibitory mode and steric hindrance in dense microenvironments (e.g., solid tumors). In highly dense solid TME, such as melanoma and NSCLC, the crowded spatial topology drives the co-localized cis-interaction between 2B4 and CD48 on the homotypic membrane of infiltrating NK cells. This cis-binding physically exerts strong steric hindrance, masking the extracellular binding domain of 2B4 and blocking its trans-binding with CD48 on tumor cells, ultimately leading to NK cell dysfunction and tumor immune escape. Abbreviations: AML, Acute myeloid leukemia; CD48, Cluster of differentiation 48; NK, Natural killer. NSCLC, Non-small cell lung cancer. TME, Tumor microenvironment. The figure was created using Figdraw (https://www.figdraw.com, accessed on 20 May 2026).

Tumor cells downregulate CD48 through multiple mechanisms to evade immune surveillance. In AML, oncogenic fusion proteins PML-RARA and acute myeloid leukemia 1-protein-eight-twenty-one protein (AML1-ETO) suppress CD48 transcription in a histone deacetylase (HDAC)—dependent manner, impairing 2B4-mediated NK cell activation [14,17]. Similarly, TGF-β within the TME downregulates CD48 expression on leukemia and solid tumor cells, reducing NK cell recognition [18]. In non-small cell lung cancer, CD48-low tumor cells exhibit stronger immune evasion, whereas CD48-positive cells enhance NK cytotoxicity [10]. Additionally, in aggressive peripheral T-cell lymphoma, malignant T cells suppress CD48 to evade both NK and T cells [18]. The immune outcome depends on four interacting axes: First, cis versus trans engagement determines whether 2B4 receives an accessible intercellular activating signal or is sequestered on the same membrane. Second, ligand density determines signal strength: insufficient CD48 may prevent NK-cell recognition, whereas excessive or persistent CD48-2B4 contact may promote inhibitory adaptor recruitment and functional adaptation. Third, the immune-cell composition of the TME determines whether CD48 mainly supports NK/CD8^+^ T-cell cytotoxicity or participates in suppressive myeloid crosstalk. Fourth, cytokines such as TGF-β, together with oncogenic and epigenetic programs, can dynamically regulate CD48 expression and thereby alter immune sensitivity over time. These variables help reconcile why low CD48 expression is immune-evasive in AML and NSCLC, whereas high CD48 expression in glioma or myeloid-rich solid tumors may correlate with immune suppression rather than effective antitumor immunity (Fig. 2).

### 2.2 Interaction between CD48 and the TME

CD48 modulates antitumor immune responses by regulating the activity, localization, and persistence of immune cells within the TME. In hepatocellular carcinoma (HCC), tumor-associated monocytes expressing high levels of CD48 induce rapid activation of NK cells, followed by their depletion, ultimately leading to NK-cell exhaustion and death. Blocking the CD48 receptor 2B4 on NK cells significantly alleviates this monocyte-induced NK-cell dysfunction [19]. In clear cell renal cell carcinoma (ccRCC), CD48 expression on tumor-infiltrating NK cells (TINKs) negatively correlates with NK-cell activation markers such as DNAM-1 and NKp30, suggesting that CD48 may contribute to an immunosuppressive microenvironment by inhibiting NK-cell function [19,20]. This dynamic regulatory mechanism indicates that CD48 may play a dual role across different tumor contexts: promoting NK-cell activation in early or permissive immune settings while potentially driving immune suppression during chronic stimulation or in myeloid-dominant niches [19]. Furthermore, in breast cancer, the co-expression of CD48 with human leukocyte antigen (HLA) class II molecules, including HLA-DOB and HLA-DQB2, enhances antigen presentation and T-cell activation, thereby promoting a synergistic antitumor immune response [2,21].

The TME also contains multiple CD48-modifying layers that should be considered when interpreting therapeutic feasibility. TAMs and myeloid-derived suppressor cells (MDSC) may provide chronic CD48/2B4 stimulation while releasing TGF-β, IL-10, prostaglandins, and arginase-dependent suppressive mediators. Hypoxia and nutrient competition can reduce NK-cell mitochondrial fitness and intensify exhaustion after checkpoint blockade. Cancer-associated fibroblasts and extracellular matrix density may further restrict immune-cell trafficking, increasing the probability of prolonged cell-cell contact without efficient target-cell lysis. Therefore, CD48-targeted therapy in solid tumors should be evaluated together with myeloid suppression, metabolic stress, hypoxia, and stromal architecture rather than as an isolated receptor-ligand intervention.

## 3 Potential of CD48 as a Target for Tumor Immunotherapy

**Figure 3:**
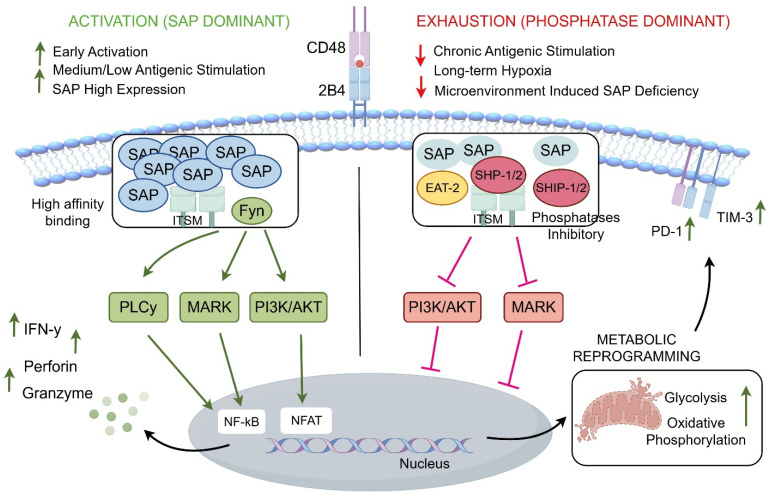
Competitive switching of intracellular adaptor proteins regulates the molecular toggle between activation and exhaustion in the CD48–2B4 axis. Left (Activation state, SAP-dominant): Under early activation or low-to-moderate antigen stimulation, SAP is highly expressed in effector cells. The trans-binding of CD48–2B4 induces the phosphorylation of ITSM within the intracellular domain of 2B4, recruiting SAP with high affinity, which sequentially recruits the Src family kinase Fyn. This initiates downstream PLCγ, MAPK, and PI3K/AKT signaling cascades, activating the transcription factors NF-κB and NFAT to promote the release of IFN-γ, perforin, and granzymes, thereby exerting anti-tumor effector functions. Right (Exhaustion state, phosphatase-dominant): Upon chronic antigen exposure, prolonged hypoxia, or microenvironment-induced SAP deficiency, the ITSM cease to recruit SAP and instead selectively bind EAT-2 or directly recruit inhibitory phosphatases (e.g., SHP-1/2, SHIP-1/2). These phosphatases negatively feedback to block the MAPK and PI3K/AKT pathways, sustaining a disruption in activation signaling. Concurrently, this triggers cellular metabolic reprogramming (with upregulation of both glycolysis and oxidative phosphorylation) and induces the expression of immune checkpoint molecules such as PD-1 and TIM-3, driving the immune cells toward functional exhaustion. Abbreviations: AKT, AK strain transforming; EAT-2, EWS/FLI1 activated transcript-2; CD48, Cluster of differentiation 48; IFN-γ, Interferon-gamma; ITSM, Immunoreceptor tyrosine-based switch motif; NK, Natural killer; NFAT, Nuclear factor of activated T cells; NF-κB, Nuclear factor kappa-light-chain-enhancer of activated B cells; PD-1, Programmed cell death protein 1; PI3K, Phosphoinositide 3-kinase; PLCγ, Phospholipase C γ; SAP, SLAM-associated protein; SHIP-1, SH2 domain-containing inositol-5-phosphatase 1; SHP-1/2, Src homology region 2 domain-containing Phosphatase; TIM-3, T-cell immunoglobulin and mucin domain-containing protein 3. The figure was created using Figdraw (https://www.figdraw.com, accessed on 20 May 2026).

The molecular switch between activation and exhaustion within the CD48-2B4 axis is schematized (Fig. 3). Based on this mechanism, several therapeutic strategies have been explored to tilt the balance toward antitumor activation.

### 3.1 Therapeutic Strategies Based on Fundamental Research

The CD48-2B4 axis is a promising immunotherapeutic target. Restoring CD48 expression—either by blocking TGF-β signaling or through epigenetic modulation (see Section 3.2.5)—can enhance NK cell-mediated antitumor activity. In ccRCC, CD48 is co-expressed with PD-1, suggesting that combining CD48-targeted agents with PD-1/PD-L1 blockade may synergistically activate both NK and T cells [20,22]. Furthermore, IL-2/IL-15 stimulation promotes homotypic 2B4-CD48 interactions between adjacent NK cells, augmenting their antitumor function [3,23].

### 3.2 Major Pharmacological Strategies Targeting CD48

#### 3.2.1 Anti-CD48 Monoclonal Antibodies

Anti-CD48 monoclonal antibodies can exert antitumor effects through multiple mechanisms. On the one hand, agonistic antibodies can directly activate the 2B4 receptor on NK cells, thereby enhancing their cytotoxic function. On the other hand, antibody-dependent cell-mediated cytotoxicity (ADCC) and CDC can also mediate direct killing of CD48-positive tumor cells [12,15]. A study by Hosen et al. demonstrated that anti-CD48 monoclonal antibodies effectively induced ADCC against MM cells *in vitro* and significantly suppressed tumor growth in a mouse xenograft model [12]. Similarly, the HuLy-m3 antibody developed by Sun et al. exhibited antitumor activity in a melanoma model [15]. However, given the broad expression of CD48 on normal hematopoietic cells, systemic administration may pose potential off-target toxicity, imposing higher demands on antibody selectivity.

A key safety concern is that agonistic stimulation may not be uniformly beneficial. In hematologic tumors with accessible CD48-positive malignant cells, antibody-mediated ADCC/CDC or restoration of NK-cell recognition may be advantageous. In compact solid tumors, however, forced CD48-2B4 activation in a dense contact environment could enhance cis-dominant or chronic inhibitory signaling, particularly when NK cells already express exhaustion markers. Accordingly, antibody design should distinguish depletion-oriented anti-CD48 antibodies from agonistic CD48-2B4 modulators and should incorporate pharmacodynamic readouts of NK-cell activation, exhaustion, and cytokine release.

#### 3.2.2 Antibody-Drug Conjugates (ADCs)

Antibody-drug conjugates achieve precise killing of tumor cells by conjugating potent cytotoxic drugs to CD48-targeting antibodies. A representative agent, SGN-CD48A, has entered preclinical development; this agent conjugates the microtubule inhibitor monomethyl auristatin E (MMAE) to an anti-CD48 antibody and demonstrates potent cytotoxic activity against tumor cells with high CD48 expression. Compared with monoclonal antibodies, the ADC strategy can reduce systemic toxicity while broadening the therapeutic window; however, its efficacy is highly dependent on the density and homogeneity of CD48 expression on the tumor cell surface.

Because CD48 is not a tumor-restricted antigen, ADC development requires stringent assessment of antigen density, internalization kinetics, payload bystander effects, and hematopoietic reserve. Heterogeneous or low-level CD48 expression may generate incomplete tumor killing, whereas expression on normal immune subsets may produce lymphopenia, myelosuppression, or prolonged immune dysregulation. These risks are especially relevant for patients previously exposed to chemotherapy or stem-cell-toxic regimens.

#### 3.2.3 Bispecific Antibodies

Bispecific antibodies can simultaneously bind CD48 on tumor cells and activating receptors on immune cells (such as T cells or NK cells), thereby directing immune cells to the tumor site. Efforts have been made to develop CD48 × CD3 bispecific antibodies, aiming to induce T cell-mediated cytotoxicity by bridging CD48-positive tumor cells and T cells. Similarly, CD48 × CD28 bispecific antibodies can further enhance T cell activation and proliferation by providing co-stimulatory signals. The advantage of this strategy lies in its ability to simultaneously engage multiple immune effector cell types; however, potential risks include cytokine release syndrome and off-target toxicity, which warrant thorough evaluation in preclinical models.

For bispecific formats, the major unresolved issue is whether CD48 provides sufficient tumor selectivity to justify immune redirection. CD48 × CD3 or CD48 × CD28 constructs may amplify T-cell activation, but they also carry theoretical risks of cytokine release syndrome, bystander activation of normal hematopoietic cells, and T-cell exhaustion under persistent antigen exposure. A more rational design may require conditional activation, affinity tuning, tumor-restricted co-targeting, or local delivery to reduce systemic immune activation.

#### 3.2.4 Engineered Immune Cells (CAR-NK/CAR-T)

CD48 can serve as a targeting antigen for the construction of CAR-modified immune cells. The K562 feeder cells expressing CD48/4-1BBL/mbIL-21 developed by Liu et al. enable efficient expansion of NK cells, providing a scalable cell source for adoptive CAR-NK therapy [7]. Furthermore, CD48-targeted CAR-T cells have also demonstrated specific killing activity against CD48-positive tumors in preclinical studies. Challenges associated with this strategy include balancing antitumor activity against normal tissue toxicity and maintaining the persistence of CAR-expressing cells *in vivo*.

For engineered-cell therapy, CD48 should be considered a high-risk target unless tumor-selective expression, safety switches, or logic-gated recognition systems are incorporated. CAR-NK strategies may have a more favorable safety profile than CAR-T approaches because of their shorter persistence and innate cytotoxic program, but the broad expression of CD48 on immune cells still raises concern for fratricide, impaired immune reconstitution, and unintended depletion of normal hematopoietic compartments.

#### 3.2.5 Epigenetic Modulating Agents

As discussed in Section 2.1, CD48 is epigenetically silenced in AML and other tumors. Hypomethylating agents such as decitabine and azacitidine restore CD48 expression, thereby re-sensitizing tumor cells to NK cell-mediated killing. Elias et al. showed that decitabine upregulates CD48 mRNA and protein in AML cells, restoring 2B4-mediated NK activation [14]. Wang et al. demonstrated that combining hypomethylating agents with adoptive NK cell transfer produces synergistic antitumor effects in AML mouse models [13]. Off-target myelosuppression remains a concern.

### 3.3 Advances in Preclinical Studies

Animal studies have validated the therapeutic potential of targeting the CD48-2B4 axis. In melanoma models, NK cells from CD48-deficient mice exhibit functional exhaustion due to impaired 2B4-mediated self-recognition, resulting in accelerated tumor progression [7]. Conversely, direct activation of this pathway using anti-CD48 monoclonal antibodies enhanced NK cell cytotoxicity against MM cells by more than threefold and significantly suppressed subcutaneous tumor growth in mice. In HCC models, TAMs promote immune evasion by inducing rapid NK cell activation followed by exhaustion via high CD48 expression. Blocking CD48–2B4 interactions partially restores NK cell function [8]. The development of novel combination strategies further expands the clinical applicability of CD48 targeting. For example, radiotherapy or chemotherapy can upregulate CD48 and NKG2D ligand expression on tumor cells, thereby enhancing NK cell infiltration and killing capacity against solid tumors [24]. In a mouse cytomegalovirus (MCMV) infection model, CD48 promoted NK cell expansion through 2B4-activated macrophages, suggesting that targeting this axis may bolster antitumor immunity [19]. Additionally, CD48–2B4 signaling regulates IFN-γ secretion and cytotoxicity by modulating NK cell–dendritic cell (DC) interactions [3,25]. Genetically engineered K562 cells expressing CD48, 4-1BBL, and mbIL-21 have also been developed as an efficient feeder layer for NK cell expansion, offering a scalable cell source for adoptive CAR-NK therapy [7].

Taken together, the preclinical evidence supports biological plausibility rather than immediate clinical readiness. Most available models evaluate short-term tumor control, NK-cell cytotoxicity, or immune-cell expansion, whereas long-term hematologic toxicity, cytokine-release potential, resistance mechanisms, and effects on immune memory remain insufficiently characterized. Future preclinical work should include humanized immune models, orthotopic solid-tumor systems, longitudinal monitoring of NK/T-cell exhaustion, and comparative testing of agonistic versus blocking strategies.

### 3.4 Potential of CD48 as a Biomarker

Pan-cancer analyses reveal heterogeneous expression of CD48 across 33 cancer types. Elevated CD48 expression is significantly associated with improved overall survival (OS) and progression-free survival (PFS) in patients with renal cell carcinoma, lung cancer, and other malignancies [2]. Within the TME, CD48 expression levels show a positive correlation with infiltration densities of CD8^+^ T cells and M1 macrophages, and a negative correlation with regulatory T cells (Tregs), suggesting its potential role as a marker of an immunologically active TME [2]. In terms of treatment prediction, CD48 expression correlates significantly with tumor mutational burden (TMB) and microsatellite instability (MSI) in 17 cancer types. Notably, in colorectal and gastric cancers, patients with high CD48 expression exhibit response rates to ICIs that are more than 40% higher than those with low expression [2]. Furthermore, a composite scoring model incorporating CD48 with other NK cell activation markers, such as NKG2D and DNAM-1, allows for more accurate prediction of immunotherapy benefit in patients with ccRCC [20].

The biomarker value of CD48 should also be interpreted cautiously. Much of the current evidence is derived from bulk transcriptomic datasets, where CD48 mRNA may reflect immune-cell infiltration rather than tumor-cell-intrinsic expression. Clinical application will require standardized assays that distinguish membrane-bound CD48 from sCD48, tumor-cell expression from immune-cell expression, and trans-activating spatial proximity from cis-inhibitory colocalization. Immunohistochemistry, flow cytometry, single-cell RNA sequencing, spatial transcriptomics, and serum sCD48 quantification may need to be integrated before CD48 can be used as a predictive biomarker for immunotherapy selection.

## 4 Challenges and Future Perspectives

### 4.1 Current Research Limitations

#### 4.1.1 Functional Heterogeneity of CD48 across Different Tumor Types

CD48 exhibits considerable functional heterogeneity in tumor immunity. For example, in AML, abnormally low CD48 expression promotes tumor immune escape by impairing recognition through the NK cell activation receptor 2B4 [14]. In glioma, however, high CD48 levels are associated with an immunosuppressive microenvironment and poor prognosis [26], whereas in melanoma, CD48 expression may attenuate antitumor immunity via inhibitory signaling pathways [27]. These context-dependent effects—either protumor or antitumor—may arise from the coordinated activity of other immune checkpoints in the TME, tumor-type-specific signaling pathways (e.g., CD48–2B4 or CD48–CD2 interactions), and variations in immune cell composition, such as the ratio of NK cells to myeloid cells. However, the underlying mechanisms remain incompletely understood [28]. Additionally, CD48 is highly expressed in certain solid tumors, including MM, where it correlates with immune cell infiltration. Although CD48 suppresses malignant transformation in hematopoietic stem cells (HSCs) through cytokine regulation (e.g., IFN-γ), it may promote proliferation of mature tumor cells by activating Pak1 signaling [29,30]. This context-dependent functional shift has yet to be fully elucidated, and the role of CD48 across other cancer types requires further validation [2,31].

A practical way to interpret these apparently conflicting findings is to separate hematologic and solid-tumor immune ecologies. In AML and MM, CD48 loss on malignant cells can directly reduce NK-cell recognition, supporting a tumor-suppressive role for CD48 expression. In glioma, melanoma, HCC, and ccRCC, CD48 expression may instead mark infiltrating immune or myeloid populations embedded in suppressive, hypoxic, and chronically stimulated TMEs. Thus, CD48 expression alone should not be equated with functional immunity; its meaning depends on cell type, ligand orientation, receptor availability, and immune-exhaustion state.

#### 4.1.2 Safety Considerations in CD48-Targeted Therapy

Although anti-CD48 monoclonal antibodies (mAbs) show significant efficacy in myeloma models without damaging normal hematopoietic stem cells, and CD48 is not expressed on red blood cells [12], potential cross-reactivity with erythrocyte surface antigens cannot be excluded. Targeted anti-CD48 therapy may also cause adverse effects such as bone marrow suppression or thrombocytopenia, attributable to the low-level expression of CD48 on hematopoietic stem cells (CD34^+^ cells) or unexpected cross-reactivity with platelets or monocytes [12,29]. Moreover, sCD48 could compromise therapeutic efficacy by neutralizing antibody activity and might even trigger autoimmune phenomena, analogous to the anemia observed with anti-CD47 antibodies. Additionally, given the broad expression of CD48 on T cells and B cells, systemic CD48 targeting carries the risk of disrupting normal immune responses [12,26].

The safety profile should therefore be evaluated at several levels: direct cytopenias caused by binding to hematopoietic cells; immune dysregulation caused by depletion or activation of T, B, NK, or myeloid subsets; cytokine release caused by immune-cell crosslinking; and impaired antimicrobial or antitumor immune surveillance after repeated dosing. These risks are not merely theoretical because CD48 is broadly expressed across leukocyte lineages, even if its expression is limited or absent on erythrocytes. Dose-escalation strategies, target-occupancy monitoring, lineage-specific flow cytometry, and serum cytokine surveillance should be incorporated into any translational development plan.

#### 4.1.3 Key Bottlenecks in Drug Development and Clinical Translation

Currently, drug development targeting CD48 remains at the preclinical stage, with no candidate having yet entered clinical trials. The primary bottlenecks include the following aspects:

(1) *Complexity of Target Biology*

The interaction between CD48 and 2B4 involves dual cis/trans binding modes, wherein simple agonism or blockade may yield unintended effects [5]. Cis interaction (binding of CD48 and 2B4 on the same cell membrane) can inhibit the functional activity of 2B4 in trans (intercellular) binding, significantly complicating the design of intervention strategies based on antibodies or small molecules. Furthermore, the interaction between CD48 and CD2 is also involved in regulating T cell activation, and targeting CD48 may simultaneously affect the function of multiple immune subsets [6].

(2) *Interference from sCD48*

sCD48 is detectable in the serum of patients with various diseases, and its levels may correlate with disease severity [32,33]. sCD48 may attenuate the efficacy of anti-CD48 monoclonal antibodies by competitively binding to 2B4 or CD2 receptors. However, the levels of sCD48 in cancer patients and its impact on immune checkpoint function have not been systematically investigated, representing a significant knowledge gap in drug development.

(3) *Expression in Normal Tissues*

CD48 is widely expressed on hematopoietic cells, including T cells, B cells, NK cells, and myeloid cells [1,2]. Although Hosen et al. reported that CD48 is not expressed on erythrocytes and that anti-CD48 monoclonal antibodies did not cause significant damage to normal hematopoietic stem cells, systemic administration may still interfere with normal immune function [12]. Additionally, weak expression of CD48 on platelets and monocytes may lead to adverse effects such as thrombocytopenia.

(4) *Lack of Reliable Animal Models*

Species differences between mice and humans exist in the expression profile of CD48 and its affinity for ligands (2B4, CD2), limiting the accuracy of preclinical efficacy evaluations. Current studies predominantly employ humanized mice or syngeneic transplantation models; however, such models cannot fully recapitulate the complex immune microenvironment in humans.

(5) *Immature Combination Therapy Strategies*

Although the synergistic effects of CD48 with classic immune checkpoints such as PD-1/PD-L1 have been mentioned in mechanistic studies [20,22], their combined application has not yet been evaluated in clinical trials. The optimal timing, dosage, and biomarker-based selection strategies for combination therapy all require systematic investigation.

### 4.2 Future Research Directions

#### 4.2.1 In-Depth Analysis of the Synergistic Mechanism between CD48 and HLA Class II Molecules

CD48 and HLA class II molecules are co-expressed on antigen-presenting cells, such as dendritic cells, and may synergistically enhance antitumor immunity by strengthening T cell co-stimulatory signaling through pathways including CD28 and CD2. For instance, combining CD48 agonists with HLA class II-restricted antigen vaccines holds promise for activating collaborative killing mediated by both CD4^+^ T cells and NK cells [3,34]. Furthermore, crosstalk between CD48 and the HLA-E/natural killer group 2 member D (NKG2D) pathway may influence the effectiveness of immune checkpoint blockade [20]. The integration of single-cell sequencing technologies to analyze the spatiotemporal distribution of cells co-expressing CD48 and HLA class II could reveal new targets for immunotherapy [2].

#### 4.2.2 Development of CD48-Targeted Combination Therapies

Gene editing targeting CD48 (e.g., CRISPR-mediated knock-in of activating CD48 variants) can enhance the recognition of tumors with low CD48 expression by CAR-T or CAR-NK cells, thereby improving the efficacy of existing immunotherapies. In combination with anti-PD-1/PD-L1 antibodies or CAR-T therapy, synergistic activation of NK cells and T cells holds promise for overcoming immunotherapy resistance. Furthermore, the use of hypomethylating agents (e.g., decitabine) to upregulate CD48 expression can restore NK cell-mediated killing activity against AML cells [13]. Additionally, bispecific antibodies designed based on the spatial interaction between CD48 and CD2 (such as CD48 × CD3 or CD48 × CD28) can specifically activate tumor-infiltrating lymphocytes [28].

#### 4.2.3 Exploring the Role of CD48 in Tumor Metabolic Regulation

The metabolic role of CD48 should be framed cautiously because direct evidence linking CD48 to tumor-cell metabolic reprogramming or mTOR-driven T-cell exhaustion remains limited. Rather than concluding that CD48 directly drives mTOR-dependent exhaustion, future studies should test whether chronic CD48-2B4 engagement alters PI3K/AKT activity, mitochondrial membrane potential, glycolytic flux, oxidative phosphorylation, lipid metabolism, and exhaustion-marker induction in NK and T cells. Particular attention should be paid to compensatory NK-cell responses after PD-1/PD-L1 blockade, because checkpoint inhibition may transiently restore effector activity while also increasing metabolic demand and susceptibility to exhaustion in CD48/2B4-rich niches. Therefore, CD48-metabolism crosstalk is best regarded as a testable hypothesis requiring direct mechanistic validation.

#### 4.2.4 Establishing a Precision Medicine-Oriented Biomarker System

Integrating CD48 expression levels, 2B4/CD48 ligand-receptor engagement, and TMB could help identify potential responders to immunotherapy. In MM, the co-expression pattern of CD48 and SLAMF7 may inform the design of bispecific antibody therapies [31]. Moreover, patients exhibiting both high CD48 expression and high TMB are likely to show enhanced sensitivity to immune checkpoint inhibition [2]. Multi-omics integrated analysis—for instance, using a triple-marker panel combining TMB, CD48, and PD-L1—could improve the prediction of immunotherapy response [2,20]. Additionally, spatial colocalization of CD48 and 2B4, as validated by fluorescence resonance energy transfer (FRET), may serve as a functional biomarker to guide rational combination therapies [3,5].

#### 4.2.5 Drug Design and Optimization Strategies Targeting CD48

Future drug development targeting CD48 should achieve breakthroughs in the following directions:

(1) *Antibody Engineering Optimization*

Enhance ADCC activity through Fc engineering (e.g., introduction of S239D/A330L/I332E mutations); alternatively, employ pH-dependent binding design to enable antibodies to preferentially bind CD48 in the acidic TME, thereby reducing exposure to normal tissues. Furthermore, the development of biparatopic antibodies can enhance affinity for CD48 and internalization efficiency, making them suitable for ADC development.

(2) *Bispecific Antibody Design*

Leverage the spatial interaction between CD48 and CD2 or 2B4 to design bispecific molecules that bridge tumor cells with T cells or NK cells. Novel bispecific T-cell engager (BiTE) or bispecific killer cell engager (BiKE) platforms hold promise for achieving potent activation at lower doses and mitigating the risk of cytokine release syndrome.

(3) *Optimization of Combination Therapy Strategies*

Systematically explore the synergistic effects of CD48-targeting agents with PD-1/L1 inhibitors, chemotherapy, radiotherapy, oncolytic viruses, and other modalities, and clarify the optimal timing, sequence, and dosage combinations through mechanistic studies. Of particular interest, the combination of CD48 agonists with ICIs may elicit more durable antitumor responses by synergistically activating innate and adaptive immunity.

(4) *Biomarker-Guided Precision Therapy*

Construct a multidimensional biomarker system based on parameters such as CD48 expression level, sCD48 concentration, 2B4 co-localization status, and TMB to identify responsive populations and improve the success rate of clinical trials. The application of single-cell sequencing and spatial transcriptomics technologies will facilitate elucidation of the spatiotemporal distribution and functional status of CD48 within the TME.

Overall, CD48 is best viewed as a context-dependent immune-regulatory hub rather than a uniformly activating target. Its therapeutic potential will depend on distinguishing membrane-bound from sCD48, trans-activating from cis-inhibitory interactions, hematologic from solid-TMEs, and validated interventions from speculative designs. A development strategy that integrates spatial biology, pharmacodynamic biomarkers, toxicity monitoring, and mechanism-based patient selection is required before CD48-directed therapies can be responsibly advanced into clinical testing.

## Data Availability

Not applicable.
